# Does *Caulerpa prolifera* with Its Bacterial Coating Represent a Promising Association for Seawater Phytoremediation of Diesel Hydrocarbons?

**DOI:** 10.3390/plants12132507

**Published:** 2023-06-30

**Authors:** Sarah Caronni, Lara A. Quaglini, Andrea Franzetti, Rodolfo Gentili, Chiara Montagnani, Sandra Citterio

**Affiliations:** Department of Earth and Environmental Sciences, University of Milano-Bicocca, Piazza della Scienza 1, 20126 Milan, Italy; l.quaglini@campus.unimib.it (L.A.Q.); andrea.franzetti@unimib.it (A.F.); rodolfo.gentili@unimib.it (R.G.); chiara.montagnani@unimib.it (C.M.); sandra.citterio@unimib.it (S.C.)

**Keywords:** phytoremediation, diesel WAF, seaweeds, *Caulerpa prolifera*, bacterial coating

## Abstract

Anthropic diesel-derived contamination of Mediterranean coastal waters is of great concern. Nature-based solutions such as phytoremediation are considered promising technologies to remove contaminants from marine environments. The aim of this work was to investigate the tolerance of the Mediterranean autochthonous seaweed *Caulerpa prolifera* (Forsskal) Lamouroux to diesel fuel and its hydrocarbon degradation potential. Changes in *C. prolifera* traits, including its associated bacterial community abundance and structure, were determined by fluorescence microscopy and next-generation sequencing techniques. Thalli of *C. prolifera* artificially exposed to increasing concentration of diesel fuel for 30 days and thalli collected from three natural sites with different levels of seawater diesel-derived hydrocarbons were analysed. Gas chromatography was applied to determine the seaweed hydrocarbon degradation potential. Overall, in controlled conditions the lower concentration of diesel (0.01%) did not affect *C. prolifera* survival and growth, whereas the higher concentration (1%) resulted in high mortality and blade damages. Similarly, only natural thalli, collected at the most polluted marine site (750 mg L^−1^), were damaged. A higher abundance of epiphytic bacteria, with a higher relative abundance of Vibrio bacteria, was positively correlated to the health status of the seaweed as well as to its diesel-degradation ability. In conclusion, *C. prolifera* tolerated and degraded moderate concentrations of seawater diesel-derived compounds, especially changing the abundance and community structure of its bacterial coating. The protection and exploitation of this autochthonous natural seaweed-bacteria symbiosis represents a useful strategy to mitigate the hydrocarbon contamination in moderate polluted Mediterranean costal environments.

## 1. Introduction

Petroleum hydrocarbon (HC) pollution from anthropic sources is an increasing problem in coastal marine areas all over the world [1,2,3,4,5]. The main potential sources of anthropogenic seawater contamination are believed to be oil spills from underground storage tanks, pipelines, land vehicles, accidental spills during transportation, drilling sites, and improper waste disposal practices [6]. According to Nikolopoulou and Kalogerakis [7], during the period between 1970 and 2007 more than 5.6 million tonnes of oil were released into the sea, as the result of both acute short-term specific events (usually large, catastrophic spills with immediate environmental effects of short duration) and prolonged small petroleum HC leaks, causing chronic exposure [8]. This is usually the case for highly urbanized spots, exposed to intense ship traffic, such as ports and harbours, but also in smaller environments [9,10]. In such chronic exposure conditions, subcellular effects including altered metabolism, cell structure and function, or the enhancement of chromosome mutations can occur in living organisms, with a cascade of biological consequences that severely affect the whole ecosystem [11]. Among petroleum HCs, one of the most common mixtures is diesel oil, utilized in both industry and daily life for energy production [12]. Diesel comprises 64% aliphatic (mostly cycloalkanes and n-alkanes), 35% aromatic and 1% olefinic compounds [13], with quite different molecular weights and solubility. The highly insoluble molecular fractions usually sink into the deeper part of the water, while the low molecular ones create a typical rainbow film, visible also to the naked eye, on the surface [14]. Moreover, the water-soluble compounds dissolve and emulsify in the water phase, resulting in a liquid medium, the soluble fraction (WSF) or water-accommodated fraction (WAF), which is more toxic than the original spilled oil [5,15,16]. Ramadass et al. [16] detected in the WAF the presence of many toxic and genotoxic compounds surpassing the safe level limits for aquatic environments, including benzene, toluene, ethylbenzene, and xylene (BTEX) and several polycyclic aromatic hydrocarbons (PAHs). Such compounds are currently listed among the most harmful and important “toxins” with a high threat potential. They can seriously affect different groups of marine organisms, among which are primary producers, likely by inhibiting photosynthesis, growth and altering biochemical pathways such as lipid production, hemolysis and glucose levels [17,18,19,20]. Due to the high risk for marine environments related to petroleum HCs and especially to diesel, several countries have implemented the legal regulation of the treatment of polluted marine waters [15]. They have started using bioremediation techniques with promising results in terms of remediation efficiency and the production of less hazardous compounds [21]. 

Bioremediation is considered one of the principal natural processes for the removal of the non-volatile fraction of oil from the environment, working also on the WAF [22]. An increasing number of studies have been published on this topic in recent decades, on both terrestrial and aquatic environments [5,23,24,25,26,27,28,29]. The basic mechanisms of bioremediation techniques are biosequestration, biodegradation, phytohydraulics, biological extraction, and volatilization, in which microorganisms and/or plants catalyse complex reactions leading to the partial or complete mineralization of environmental organic contaminants [30]. In particular, hydrocarbon-oxidising bacteria (HOB) have the ability to break down or transform the chemicals of petroleum products [31,32,33,34], and their association with plant organisms seems to increase the remediation power [35,36,37,38,39]. Besides the synthesizing enzymes playing an important role in HC degradation, plants also supply the indigenous microbial population with different exudate compounds, enhancing their growth and/or favouring specific useful strains [40]. On the other hand, bacteria possessing hydrocarbon-degradation pathways and metabolic activities remarkably improve plant tolerance to such pollutants [39,41,42,43]. Additionally, they can fix atmospheric nitrogen, supplying it to plants, and synthesize several different compounds that can enhance various stages of plant growth [39,44,45].

When considering the marine environment, algae, more than seagrasses, have been reported to be able to use and accumulate various contaminants, including hydrocarbons, and, due to their fast growth rates and simple nutrient requirements, they are considered a promising group of marine organisms for bioremediation [46,47]. Nevertheless, only a few papers on the HC degradation potential of algae are available in the literature, and most of them relate to microalgae (phytoplankton). The few dealing with macroalgae consider a very limited number of species, including mainly *Ulva lactuca* [4,5,48].

Recently, some *Caulerpa* species have offered promising results in seawater bioremediation [49]. *Caulerpa* spp. form a large and variegate group of green siphonous macroalgae widely distributed across tropical and temperate latitudes and abundant also in the Mediterranean Sea [50]. *Caulerpaceae* yielded interesting results, especially in the treatment of seawater from aquaculture [51,52]. Nevertheless, no data are available in the literature on the bioremediation potential of *Caulerpa* species in response to hydrocarbon pollution. Interestingly, some *Caulerpaceae* and, in particular *Caulerpa prolifera* (Forsskål) Lamouroux, which is autochthonous to the Mediterranean, are described as quite abundant in ports and harbours [53,54] frequently affected by chronic HC pollution. 

The aim of this work was to investigate the role of *C. prolifera* in seawater remediation of coastal marine environments contaminated by diesel-derived hydrocarbons. An experiment in controlled conditions was carried out to test the tolerance of *C. prolifera* with its associated bacteria to different diesel oil concentrations and to evaluate the whole degradative ability of the above-mentioned association. Changes in seaweed traits, in the abundance and structure of the bacterial community associated with the fronds and in HC water concentration were determined. Data on the microbial community abundance and structure were also compared with those of *C. prolifera* populations collected in marine sites with different concentrations of seawater diesel oil. 

## 2. Results

### 2.1. Diesel Exposition of C. prolifera in Controlled Conditions

#### 2.1.1. *C. prolifera* Functional Traits

**Figure 1:**
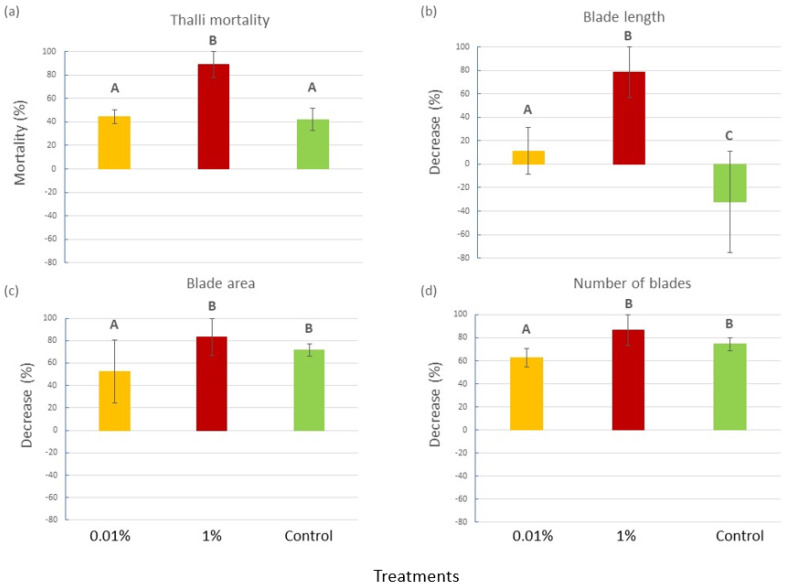
(**a**) Thalli mortality (mean percentage ± SE), (**b**) decrease in blade length (mean percentage ± SE), (**c**) blade area (mean percentage ± SE) and (**d**) blade number (±SE) for each experimental treatment (0.01% *v*/*v* diesel, 1% *v*/*v* diesel and Control with no diesel). Upper case letters indicate statistically significant differences between treatments (P < 0.05).

By the end of the 4-week experiment, *C. prolifera* thalli in the 1% *v*/*v* diesel tanks showed a significantly higher mortality (89%) than the thalli in the Control and 0.01% *v*/*v* diesel tanks (42 and 44%, respectively; Figure 1a), as confirmed by the ANOVA (P = 0.0189) and the SNK post hoc tests (Table S1 in the Supplementary Materials). Moreover, for almost all of the traits measured on the living thalli, a general decreasing trend was observed throughout the experimental time, and it appeared to be significantly more pronounced for the 1% tanks than in the other treatments, for which different situations were observed in relation to the considered trait (Figure 1b–d). In particular, a similar decrease in the mean blade number and area was observed for the Control and 1% treatment, while it was significantly less pronounced for the 0.01% *v*/*v* one (Figure 1b–d and Table S2 in the Supplementary Materials). In contrast, for the mean blade length, significant differences were recorded among all the treatments. In particular, in the Controls, the blade length increased, whereas a consistent and a small decrease were observed in the 1% and 0.01% treatments, respectively (Figure 1b–d and Table S2 in the Supplementary Materials).

#### 2.1.2. Bacterial Abundance on *C. prolifera* Blades 

**Figure 2:**
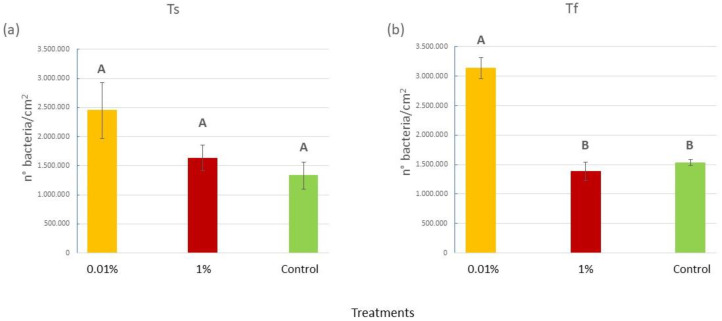
(**a**) Mean (±SE) epiphytic bacteria abundances (expressed as number of bacteria/cm^2^) for the three treatments (0.01% *v*/*v* diesel; 1% *v*/*v* diesel; Control with no diesel) at the beginning of the experiment (T_s_); (**b**) mean (±SE) epiphytic bacteria abundances (expressed as number of bacteria/cm^2^) for the three treatments (0.01% *v*/*v* diesel; 1% *v*/*v* diesel; Control with no diesel) at the end (T_f_) of the experiment. Upper case letters were used to indicate statistically significant differences between the treatments at T_s_ and T_f_ (P < 0.05).

At the beginning of the experiment, the number of epiphytic bacteria on the blades of *C. prolifera* did not change among treatments, while some significant differences were observed overall at T_f_ (Figure 2), as confirmed by the ANOVA (P = 0.0002) and the SNK post hoc test (Figure 2a,b and Table S3 in the Supplementary Materials). Notably, algae in tanks with 0.01% *v*/*v* of diesel hosted more than double the number of bacteria (3.1 × 10^6^ ± 0.09 × 10^6^ bacteria/cm^2^) of the algae in the 1% and Control tanks (1.4 × 10^6^ ± 0.007 × 10^6^ and 1.5 × 10^6^ ± 0.06 × 10^6^ bacteria/cm^2^, respectively). 

#### 2.1.3. Characterization of Bacterial Communities 

**Figure 3:**
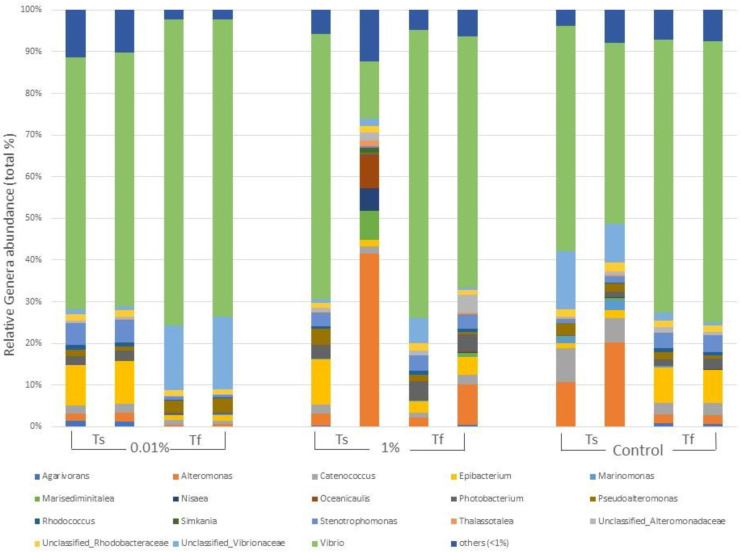
Main genera forming the epiphytic bacterial communities of *C. prolifera* at the beginning (T_s_) and at the end (T_f_) of the experiment for each treatment (0.01% *v*/*v* diesel, 1% *v*/*v* diesel, Control with no diesel). Each genus is expressed as relative abundance (%) of the total genera in the sample. Genera whose abundance was <1% were grouped in “others”.

The genera composition of the bacterial epiphytic community for each treatment at the beginning and at the end of the experiment and differences among them are reported in Figure 3. OTUs assigned to the genus *Vibrio* accounted for 62.78% of total diversity, for both samples at T_s_ and at T_f_. However, their abundance varied among treatments; in particular, by the end of the experiment, the epiphytic community in 0.01% tanks were characterized by a higher percentage of OTUs relative to *Vibrio* (72.43%) than the community of the other treatments (64.72% in 1% and 66.48% in Control; Figure 3). 

**Figure 4:**
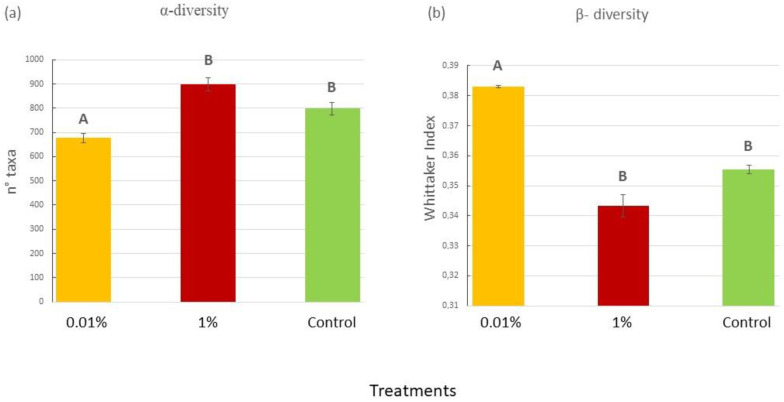
(**a**) Mean (±SE) number of bacterial taxa at T_f_ in the experimental samples for each treatment (0.01% *v*/*v* diesel; 1% *v*/*v* diesel; Control with no diesel); (**b**) Whittaker index at T_f_ in the experimental samples for each treatment (0.01% *v*/*v* diesel; 1% *v*/*v* diesel; Control with no diesel). Data for T_s_ are not shown, since no significant difference was found by the ANOVA analyses. Upper case letters indicate statistically significant differences between treatments (P < 0.05).

At the beginning of the experiment, the OTU richness index did not differ among treatments (P = 0.055). By the end of the experiment, however, the ANOVA highlighted a statistically significant difference between treatments (Figure 4a; P = 0.0494). In particular, the 0.01% treatment showed the lowest number of OTUs in comparison with the other treatments (Table S4 in Supplementary Materials). The Whittaker index, used as a measure of β-diversity between samples, did not differ among treatments at the start of the experiment (Table S5 in the Supplementary Materials), but, by the end, it significantly increased in the 0.01% samples if compared to the other treatments (P = 0.0066), as shown in Figure 4b and in Table S5 of the Supplementary Materials. 

**Figure 5:**
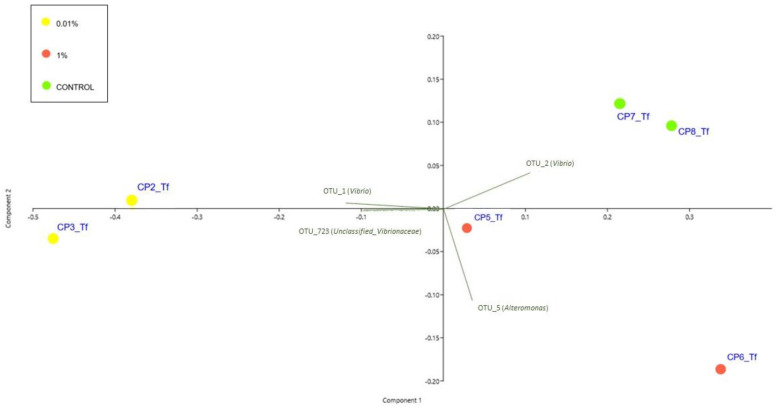
PCA biplot on Hellinger-transformed abundances of each OTU of T_f_ experimental samples. The plot also shows the principal bacterial taxa that mostly contribute to the patterns observed. Symbol colours for the treatment: yellow dots stand for 0.01% diesel concentrations; red dots stand for 1% diesel concentrations; green dots stand for Controls. T_s_ experimental samples were not included in the biplot.

The PCA (Figure 5) showed that samples at T_f_ clustered in two groups: the 0.01% were spatially disposed on one side of the first principal component (PC1) while all the other samples lay on the opposite side. Figure 5 also reports the principal bacterial OTUs that contribute to the observed patterns. The trend highlighted by the PCA was confirmed also by the PERMANOVA, which gave no significant results for T_s_ (data not included in the biplot) while highlighting the significance of differences observed at T_f_ (P = 0.021) (Figure 5 and Table S10 in the Supplementary Materials). The SIMPLER post hoc test helped to define the relative contribution of different OTUs to the dissimilarities observed at T_f_. In particular, the comparison between the 0.01 and the 1% samples showed an average dissimilarity of 43.47%, and the OTUs that mostly contributed to this dissimilarity were OTU_1 (*Vibrio*) (4.97%), OTU_2 (*Vibrio*) (4.62%), OTU_723 (*Unclassified_Vibrionaceae*) (3.77%) and OTU 5 (*Alteromonas*) (2.04%). Comparing the 0.01% and Control samples, the average dissimilarity was, instead, 47.48%, and it appeared to be mostly caused, once again, by OTU_1 (*Vibrio*) (4.82%), OTU_2 (*Vibrio*) (4.59%), OUT_723 (*Unclassified_Vibrionaceae*) (4.06%) and OTU_3 (*Epibacterium*) (2.31%). Lastly, the 1%–Control sample comparison showed an average dissimilarity of 27.32%, mostly caused by OUT_3 (*Epibacterium*) (1.97%), OTU_723 (*Unclassified_Vibrionaceae*) (1.78%), *Vibrio2* OTU_1 (*Vibrio*) (1.75%) and OUT_5 (*Alteromonas*) (1.64%). 

Finally, the ANOVAs and the SNK post hoc tests revealed that the OTU_1 and OTU_2 (both assigned to the genus *Vibrio*) differed significantly between treatments (P = 0.0301 and P = 0.0114, respectively), with OTU_1 more abundant in the 0.01% samples and OTU_2 in the Control and 1% samples (0.01% < 1 = Control) (Figure 3 and Table S6 in the Supplementary Materials). 

#### 2.1.4. Water Diesel-Derived Hydrocarbon Degradation

**Figure 6:**
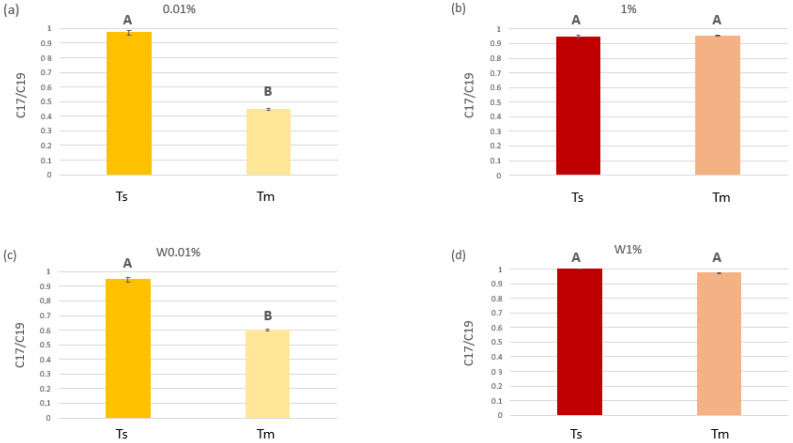
C17/C19 ratios measured at the beginning (T_s_) and at the middle of the experiment (T_m_). (**a**) and (**b**) refers to tanks with 0.01% and 1% *v/v* diesel, respectively, where *C. prolifera* was present. (**c**) and (**d**) refers to control tanks with 0.01% and 1% *v/v* diesel, respectively, where *C. prolifera* was missing. Upper case letters indicate the results of the *t*-test (P < 0.05) performed to check for statistical differences in diesel concentrations at the beginning (T_s_) and at the middle (T_m_) of the experiment, for each treatment (the two diesel concentrations and the presence/absence of the algae).

The n-C17/pristane ratio varied significantly among diesel treatments as well as between the two sampling times, in relation to the presence of *C. prolifera* in the tanks, as confirmed by the different statistical analyses performed on the data (Figure 6 and Table S7 the Supplementary Materials). Moreover, according to SNK results, for both diesel concentrations, the n-C17/pristane ratio was lower when *C. prolifera* was present (Table S7 in the Supplementary Materials); the lowest statistically significant values at Tm were recorded in tanks with *C. prolifera* and the 0.01 *v*/*v* of diesel (Figure 6a). 

### 2.2. Natural Exposure of C. prolifera to Diesel Fuel in Marine Environments

#### 2.2.1. Bacterial Abundance

**Figure 7:**
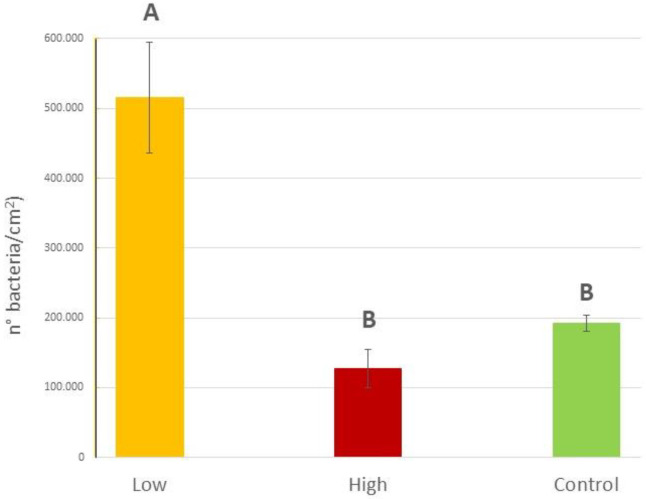
Epiphytic bacteria abundances (expressed as number of bacteria/cm^2^) on *C. prolifera* thalli collected in the field at the three different sites with different diesel concentrations (low: 84.38 mg L^−1^, high 730.57 mg L^−1^ and a Control: 31.99 mg L^−1^). Upper case letters indicate statistically significant differences between field samples (P < 0.01).

When exploring the epiphytic bacterial abundance on blades of *C. prolifera* collected in sites with different diesel contamination levels, statistically significant differences were found (P = 0.0017). Specifically, on *C. prolifera* thalli collected in the low diesel concentration site (Isola Piana, 84.38 mg L^−1^) the epiphytic bacterial abundance was more than 4 times higher than that recorded at the higher concentration (Cala Finanza, 730.57 mg L^−1^; 0.51 × 10^6^ ± 0.08 × 10^6^ bacteria/cm^2^ vs. 0.13 × 10^6^ ± 0.03 × 10^6^ bacteria/cm^2^) and 2.5 times higher than that on algae in the Control site (Punta Don Diego, 31.99 mg L^−1^; 0.19 × 10^6^ ± 0.01 × 10^6^ bacteria/cm^2^) (Figure 7), as confirmed also by the SNK post hoc test (Table S8 in the Supplementary Materials). 

#### 2.2.2. Characterization of Bacterial Communities 

**Figure 8:**
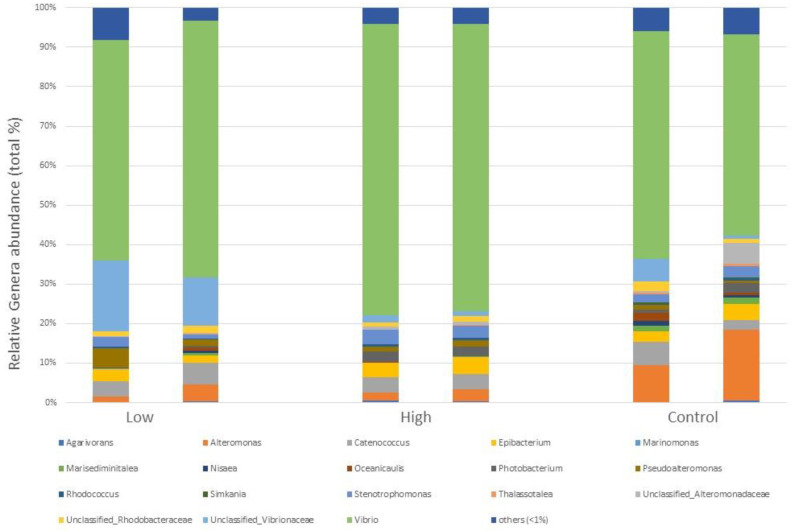
Main genera forming the epiphytic bacterial communities of *C. prolifera* collected in the field in three different locations, corresponding to different hydrocarbon (oil) contamination levels (low: 84.38 mg L^−1^, high 730.57 mg L^−1^ and a Control: 31.99 mg L^−1^). Each genus is expressed as relative abundance (%) of the total genera in the sample. Genera whose abundance was <1% were grouped in “others”.

The composition of the bacterial communities considering OTUs assigned to the different genera in the three study sites and the differences among them are reported in detail in Figure 8. OTUs assigned to the genus *Vibrio* accounted, once again (cfr. 2.1.3), for 62.87% of total diversity, although their abundancy was higher in the high-contamination site (73.30%) and lower in the low-contamination and Control sites (60.41% and 54.29%, respectively) (Figure 8). 

Both the α-diversity (expressed as number of OTUs) and the β-diversity (expressed with the Whittaker index) within the field-collected samples did not differ among sites, although for the latter the *p*-value turned out to be near significance (P_α-diversity_ = 0.194; P_β-diversity_ = 0.0638; Table S9 in the Supplementary Materials).

**Figure 9:**
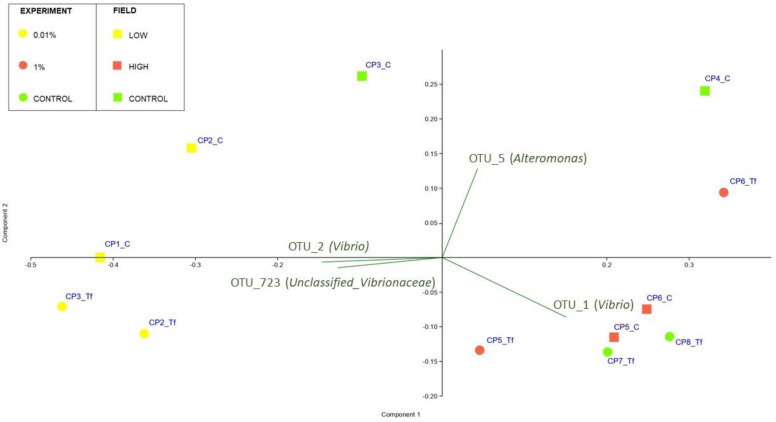
PCA biplot on Hellinger-transformed abundances of each OTU of T_f_ experimental and field samples. The plot also shows the principal bacterial taxa that mostly contribute to the patterns observed. Symbol colours and shapes denote the treatment/site: round stands for experimental samples; square stands for field-collected samples. Yellow stands for Low/0.01% diesel concentrations; red stands for High/1% diesel concentrations; green stands for Controls. Ts experimental samples were not included in the biplot.

When analysing the results of the PCA analyses, the pattern is not as clear as that obtained with laboratory samples (Figure 9), although the low-contaminated site samples cluster together with the 0.01% T_f_ samples. Contrary to what was observed for the T_f_ experimental samples, the PERMANOVA analyses gave no significant results for the field-collected samples (Table S10 in the Supplementary Materials). 

## 3. Discussion

The obtained results provide interesting information regarding both the tolerance of *C. prolifera* with its bacterial coat to diesel fuel and its hydrocarbon degradation potential.

Firstly, by analysing data regarding the functional traits of *C. prolifera* thalli, it was evident that additional factors to diesel played a role in the survival and growth of algae during the experiment in controlled conditions. As expected on the basis of the literature [55,56], the tank conditions (mainly limited water recycle and exchange) affected the health of the algae, as only about 60% of the Control thalli survived and the remaining showed a reduction in both the area and the number of blades after 30 days (the end of the experiment). Nonetheless, as all the tanks were kept at the same conditions except for the concentration of diesel, tank effects were expected to be similar for all the treatments and not to affect the different algal responses to diesel observed among them. 

The comparison between treatments and Controls highlighted statistical differences due to diesel exposition. Overall, the lower concentration of diesel (0.01%) did not affect *C. prolifera* survival and growth, whereas the higher concentration (1%) resulted in high mortality and blade damage. Several studies have proved that the presence of petroleum in seawater is an oxidative stress factor for most organisms, including algae, affecting their survival and growth, to which the more tolerant ones usually respond by activating protective and recovery mechanisms such as the production of antioxidant enzymes. Accordingly, it is possible to speculate that petroleum induced an oxidative stress also in *C. prolifera*, which was able to cope with it only at relatively low concentrations. Indeed, in comparison with the Control, a significantly higher thallus mortality was recorded only for *C. prolifera* exposed to 1% (*v*/*v*) diesel. Moreover, in this treatment only, the living algae showed a decrease (%) in all the considered traits. The behaviour of the algae exposed to 0.01% diesel was similar to that of the Control, and they even showed a significantly lower percentage decrease in blade area and number.

These results are in accordance with those of [20], who reported that, under WAF stress, macroalgae survival and growth were maintained and, in some cases, also promoted low hydrocarbon concentrations, while some more marked negative effects occurred at higher concentrations. At high concentrations, [20] showed that WAF inhibits macroalgal growth through changes mainly involving their photosynthetic activity. According to [57], these alterations are not a direct effect of toxicity, but the results of the physical effect of the film of diesel that directly settles on algae growing in quite shallow waters contaminated by oil. The effect of such film interests all macroalgae, independently of their tolerance to WAF toxicity, reducing their growth and, in case of high diesel concentrations, increasing their mortality. Indeed, the film could persist on the alga surface for a long time, even with active flushing, causing a reduction in gas exchange, nutrient uptake, and metabolite withdrawal, which directly influences the intensity of photosynthesis [58]. This seems to be particularly true especially for green algae like *C. prolifera*, which, being mostly C-3 plants [59], cannot store carbon in intermediate compounds. Moreover, oil products can lead to the inhibition of PS II with a significant reduction in the quantum yield of photosynthesis [60]. The adhesion strength and, accordingly, the period of film–alga contact depends not only on the type of oil product and on the film thickness [61], but also on the structure of the algal thallus and on the area of the exposed surface. For species with a complex organisation of the thallus, especially if syphonocladal, such as *C. prolifera*, the film of diesel will be retained for an order of magnitude longer [57]. According to our laboratory results, the physical effect of diesel film significantly affected *C. prolifera* only when the diesel concentration in the water was particularly high (1% *v*/*v*), as already observed for other species of green algae [20]. 

Furthermore, in addition to the ability to minimize oxidative stress by synthesizing antioxidant enzymes and to resist the physical effect of diesel film on their blades, macroalgae are able to tolerate diesel through their bacterial coating, by modifying the abundance and species composition of their associated bacterial community. Indeed, when diesel concentrations are not so high as to be lethal, macroalgae excrete different metabolic bioproducts in response to oxidative stress [5]. Some recent studies have demonstrated that such substances likely contribute to the development and growth of a complex and differentiated microbial community associated with the algae, especially living on their blades as epiphytes, representing their bacterial coat. Specifically, several studies have reported a significant change in both bacterial abundance and, in some cases, also in the bacterial community structure in the presence of crude oil in superficial coastal areas. In particular, the presence of different taxa of hydrocarbon-oxidizing bacteria (HOB) has been reported (e.g., [62,63]). Moreover, beside taxa specialised in hydrocarbon degradation, such as, for the marine environment, *Arthrobacter*, *Rhodococcus* and some species of the *Vibrio* genus [64,65,66], the ability to degrade petroleum hydrocarbons is almost ubiquitous among marine bacteria [23,67].

Among oil products, diesel, due to its composition, represents an excellent substratum for HOB proliferation [68]. Usually, in the case of the presence of diesel, HOB find the right conditions to proliferate, especially on seaweed blades, forming a mutually beneficial symbiotic association with them [69]. HOB are indeed able to degrade most of the components of polycyclic aromatic hydrocarbons contained in the WAF, generating other less toxic metabolites that can be absorbed and included by the algae themselves [48]. The presence of such bacteria on the seaweed blades also reduces the thickness of the diesel film, favouring algal survival and thus enhancing the production of bioactive compounds, necessary for bacteria development. For this reason, HOB living in association with algae can significantly biodegrade a higher quantity of crude oil composts when compared to free-living bacteria in seawater [70]. According to [71], hydrocarbon-oxidizing bacteria are widely distributed in nature, since hydrocarbons, even if pollutants, are anyway natural products. However, although HOB are normally present in all natural areas, their concentration is highly variable. Clearly, the presence of hydrocarbons in the environment is the principal factor that brings a selective enrichment in situ for hydrocarbon-utilizing microorganisms, especially when macroalgae capable of successfully addressing WAF toxicity are present. However, the ratio of hydrocarbon-oxidizing bacteria to the total population of heterotrophic bacteria, as well as the variety of hydrocarbon-degrading microorganisms, may change according to several factors related to the environmental conditions and the particular WAF composition [62].

At the end of the experiment described here, we observed a significantly higher epiphytic bacterial abundance in tanks with 0.01% *v*/*v* of diesel than in the Controls and 1% diesel concentration. Comparing these results with those obtained for other macroalgae, such as *Fucus vesciculosus* and *Acrosiphonia arcta*, a different trend was observed, as for such species the density of epiphytic bacteria (especially HOB) on the thalli of the seaweeds increased during the experimental period in all treatments with diesel, independently of its concentration, and varied between 0.86 mg L^−1^ and 86,000 mg L^−1^ [5,72]. However, these differences in the observed patterns can be explained considering the experiment length and conditions and the different behaviour toward diesel fuel of each species, as already observed by [73]. The duration of our experiment was significantly longer (30 days vs. 10 days), although we observed a significant worsening of the algal health status from the beginning of the exposition with the higher diesel concentration (1%), thus suggesting a minor tolerance of *C. prolifera* than *Fucus vesciculosus* and *Acrosiphonia arcta* to diesel fuel. Interestingly, in our experiment the bacterial abundance on *C. prolifera* blades was related to the health status of the seaweed. 

It is possible to speculate that, when the diesel concentration is not so high as to affect the survival of *C. prolifera*, the species actively responds to the oxidative oil-related stress with the production of compounds that enhance the development of a rich bacterial community, which in turn contributes to oil degradation. Conversely, when the diesel concentration in seawater is too high to allow *C. prolifera* and its initial bacterial coating to cope with it, the worsening health status of the algae results in the failure of epiphytic bacteria to increase in number.

In such conditions, indeed, a significant decline in the thalli rapidly occurs and, consequently, none of the cellular mechanisms that lead to the excretion of the compounds necessary for the enrichment of the bacterial community can be activated. This pattern seems to be confirmed also by the results from field *C. prolifera* thalli. A significantly higher abundance of microorganisms was, indeed, recorded on the healthy blades of *C. prolifera* collected at Isola Piana, where, according to the results of the GC analysis, diesel was present in the water in a lower concentration than at Cala Finanza (84.38 mg L^−1^ vs. 730.57 mg L^−1^), where the algae appeared to be suffering at the time of sampling. 

Moreover, data regarding the biodegradation activity by *C. prolifera* in relation to the different tested diesel concentrations suggested that the more abundant microbial community observed on algal blades was effectively composed of HOB taxa. Indeed, the lowest values for the nC17/pristane ratio were recorded in tanks with *C. prolifera* and 0.01% diesel *v*/*v* thus proving that in such conditions the biodegradation activity by the alga with its microbial community was particularly intense. On the contrary, a significantly higher C17/pristane ratio was obtained for the same diesel concentration in tanks without *C. prolifera* showing the active role of the bacteria–alga association in diesel degradation. 

Diesel oil degradation was expected to occur both in tanks with and without *C. prolifera*, as it is performed not only by the association between the macroalga and its bacterial coat but also by HOB bacteria freely living in the seawater [70]. For this reason, the degradation took place also in tanks without *C. prolifera*, but it was enhanced by the seaweed presence, as shown by the statistical analysis (Table S7). Likely, the same occurred also in tanks with 1% diesel; however, it was not evident, due to the higher HC concentration, which made degradation more difficult and less appreciable both in tanks with and without *C. prolifera* thalli.

These last results proved that *C. prolifera* played an active role in degrading diesel hydrocarbons, likely including the selection of HOB taxa that were highly represented in the epiphytic bacterial community recorded in tanks with 0.01% *v*/*v* of diesel. This hypothesis is supported by the results we obtained for the bacterial community structure analysis on *C. prolifera* blades exposed to diesel in artificial or natural environments. It is known that in natural conditions, the richness of the bacterial community usually increases markedly on unhealthy thalli [74], due to the decrease in the concentration of the alga defence compounds. The loss of the selective and inhibitory effect of the algae chemical defences allows for a greater variety of bacteria to colonize them [75]. Therefore, based on the literature, we expected to find a higher α-diversity and a similar or higher β-diversity for the most contaminated conditions. On the contrary, we found a higher α-diversity but a lower β-diversity for samples artificially exposed to the higher diesel concentrations, whereas we found a lower α-diversity and a higher β-diversity for samples exposed to lower concentrations. Taken together, α- and β-diversity suggest that at lower diesel concentrations the epiphytic bacterial community was more homogeneous, but with a different structure from both that of the Controls and of the seaweeds exposed to higher diesel concentrations. The different taxon composition of the epiphytic communities was confirmed by the PCA scatter-plot results, which clearly distinguished natural and laboratory samples exposed to lower diesel concentrations from the Controls and the higher diesel concentration-exposed samples. 

According to statistical analysis, bacterial OTUs assigned to *Vibrio* and *Unclassified Vibrionaceae* were the main taxa responsible for the differences among samples. Even if some members of the genus *Vibrio* have been described as the main etiological agents of diseases affecting humans and marine organisms, some other species are necessary for driving fundamental ecosystem processes, such as the carbon cycle and osmoregulation [76]. Moreover, some bacteria belonging to the genus *Vibrio* are included among HOB with a significantly high ability to degrade diesel and the WAF it produces in the marine environment (e.g., [77,78,79]). 

Taking into account the natural presence of *Vibrio* bacteria on *C. prolifera* blades during summer [80,81,82,83,84], their increased amount observed in relation to diesel contamination in such periods of the year suggests that they are actively involved in diesel degradation. However, only at relatively low concentrations of diesel can *C. prolifera* efficiently select and induce the proliferation of diesel-degrading *Vibrio* species, taking advantage of them in terms of tolerance. At higher concentrations, while surviving in the wild but showing less growth and blade damage in the same way as when exposed to artificial conditions, the alga is likely too stressed by diesel fuel to stimulate the growth of specific bacteria strains that efficiently degrade hydrocarbons.

## 4. Materials and Methods

### 4.1. Experimental Design 

About 150 thalli of *C. prolifera* were collected in late spring 2021, along the coasts of Tavolara Punta Coda Cavallo Marine Protected area (north-east Sardinia), in the small harbour of Cala Finanza (40°52.759′ N; long: 9°38.759′ E). During collection, the main environmental parameters (seawater temperature, salinity and N and P concentrations) were measured, according to the sampling methods described in [85]. After collection, the thalli were kept in seawater and immediately transported to the laboratory. Once there, they were transplanted in 9 sterile transparent aerated plastic tanks with subsand filters (15 thalli in each), filled in with 10 L of seawater collected in an uncontaminated area of Bergeggi Marine Protected Area (A zone), along the Liguria coasts (44°23.417′ N; 8°98.400′ E). A thin layer (1 cm) of fine sterilized sand (1 mm) was prepared as substratum for the settlement of the algae; sterilization was achieved by autoclaving the substratum at 120 °C and 1 atm for 20 min [85]. All the tanks were initially maintained in a growth chamber for 2 weeks to favour their acclimation. Field environmental conditions were simulated in each tank and controlled until the beginning of the experiment. In particular, salinity and N and P concentrations were adjusted to 35 psu, 0.7 and 0.9 mM, respectively, and a day cycle of 12 h of light at 27 °C and 12 h of dark at 23 °C was set up. During this period, *C. prolifera* thalli remained healthy in the tanks, and their health status was evaluated by considering the colour intensity of the blades, the presence of injuries, and the damaged portions, according to [86]. After acclimation, the thalli were exposed to a “low” (0.01% *v*/*v* 83.5 mg L^−1^) and a “high” diesel oil concentration (1% *v*/*v* 8350 mg L^−1^). A control with *C. prolifera*-acclimatized thalli without diesel addition was also set up. In this way, the most frequent conditions in the ports and harbours of the Mediterranean Sea were simulated (according to [1,4,5]). For each treatment, three replicated tanks were prepared. In addition, a set of control tanks without *C. prolifera* were installed for each condition (Water Controls: W0.01%; W1%; WControl), according to a fully orthogonal experimental design (*n* = 3, Figure 1). After diesel addition, WAF formation, favoured by aeration that allows soluble hydrocarbons to be transferred from the diesel film to the aqueous phase [87], was verified using gas chromatography (see below, in the text). The experiment started after acclimation (T_s_) and lasted 4 weeks (T_f_), during which time all the tanks were kept in the same condition, except for the concentration of diesel. At the end of the experiment, changes in plant traits and in the amount and structure of the algae epiphytic bacteria were determined. The biodegradation of water hydrocarbons was also estimated after 2 weeks from the beginning of the experiment (T_m_), following the procedure explained later in the text.

In parallel to the analysis of *C prolifera* exposed to HC in controlled conditions, naturally exposed thalli were collected and analysed. Specifically, about 10 thalli of *C. prolifera* were collected in the second part of August 2022 along the coasts of Tavolara Punta Coda Cavallo Marine Protected area (north-east Sardinia). Thalli were collected in the following three selected sites, characterized by quite different boat traffic and, therefore, a presumed different diesel water concentration: Punta Don Diego (the control site), where boat traffic was almost completely absent (40°487.628′ N; 9°65.588′ E), Isola Piana (40°88.651′ N; 9°64.629′ E) with low boat traffic, and Cala Finanza harbour (40°52.759′ N; long: 9°38.759′ E), with high boat traffic. After collection, the thalli were kept in seawater and immediately transported to the laboratory for the subsequent analyses.

### 4.2. C. prolifera Functional Traits

The following *C. prolifera* functional traits were measured on at least 3 thalli from each tank: number of blades, total blade length (cm) and total blade area (cm^2^). Measurements were carried out by image analysis (ImageJ, version 1.53) on pictures taken at the beginning (T_s_) and at the end of the experiment (T_f_). The mortality in terms of the number of dead thalli in each tank was also calculated at the end of the experiment.

### 4.3. Algae-Associated Bacteria Counts

At the beginning (T_s_) and at the end (T_f_) of the experiment, 2 healthy blades belonging to different thalli in each tank were put in 50 mL sterile centrifuge tubes filled with sterile (autoclaved) seawater, and additionally passed through a 0.2 µm pore size filter, prior to use. To remove non-attached microorganisms, blades were treated according to the procedure suggested by [88], modified as following. Each blade sample was rinsed on both sides with a 10 mL stream of filtered seawater, aseptically. The rinsed samples were placed in empty sterile petri dishes and vigorously swabbed on both sides with a sterile cotton-tipped applicator. Swab tips were then placed in 15 mL micro centrifuge sterile tubes containing 0.5 mL of filtered seawater and vortex-mixed for 30 s. Finally, the applicator tips were removed from the tubes and the resulting bacterial suspensions were vigorously hand-shaken and diluted (1:4) with filtered seawater to obtain a final suspension volume of 2 mL. Total bacterial counts (number of bacteria/cm^2^) were carried out by using epifluorescence microscopy and 4’, 6-diamidino-2-phenylindole (DAPI) staining, according to standard procedures [89]. Briefly, filtered (0.2 µm) DAPI solution was added to the suspensions (at the final concentration of 2 µg ml^−1^) and incubated for 10 min in the dark. The bacteria were observed with a Zaiss Axioplan microscope and a Bürker counting chamber was used to enumerate the bacteria. Counts were expressed as the number of bacteria for 1 cm^2^ of blade area.

### 4.4. Bacterial Analysis Using High-Throughput Sequencing

Bacterial DNA extraction and amplification, as well as sequence processing, were performed according to [90]. Total DNA was extracted from 1.5 mL of each bacterial suspension (kept at −20 °C) with the FastDNA^®^ Spin for Soil kit (MP Biomedicals, Solon, OH, USA), according to the manufacturer’s instructions. A first PCR amplification was performed on the V5–V6 hypervariable regions of the 16S rRNA gene for each sample, to evaluate its quality on the original and on the 1:10 and 1:100 dilutions to identify inhibition or insufficient samples. A second PCR was then performed with GoTaq^®^ Green Master Mix (Promega Corporation, Madison, WI, USA) and 1 μM of each primer, for a final volume of 2 × 50 μL for each sample. Customized oligonucleotide barcodes (6 bp) were added at the 5′ end; 783F and 1046R primers were used [91,92], and the cycling conditions were initial denaturation at 94 °C for 4 min, 28 cycles at 94 °C for 50 s, 47 °C for 30 s, 72 °C for 45 s, and a final extension at 72 °C for 5 min. The amplicons were then purified using the Wizard^®^ SV Gel and PCR Clean-up System (Promega Corporation, Madison, WI, USA) and quantified with Qubit^®^ (Life Technologies, Carlsbad, CA, USA). Libraries were prepared with nine samples each, identifiable thanks to different barcode pairs. Library preparation with the addition of standard Nextera indexes (Illumina, Inc., San Diego, CA, USA) and sequencing with the MiSeq Illumina platform (Illumina, Inc., San Diego, CA, USA), using a 2 × 300 bp paired-end protocol, was performed. The obtained reads were demultiplexed according to the indexes and barcodes. The Uparse pipeline was used for the following elaborations [93]. Forward and reverse reads were merged only if there were zero mismatches, and quality filtered with default parameters. Operational taxonomic units (OTUs) were defined with an aggregative clustering of sequences with 97% of sequence identity. Suspected chimeras and singleton sequences (i.e., sequences appearing only once in the whole dataset) were removed. Singletons (OTUs present once in one sample only) were removed from the analyses, because their inclusion could inflate the variance explained by multivariate analyses [94]. OTU classification at order and genus level was inferred using the RDP classifier [95]. To compare diversity among samples that largely differed in the number of sequences, 20,000 sequences were randomly selected from each sample for which more than 20,000 sequences were available. For the other samples, OTU abundance was normalized to 20,000 sequences by resampling with repetition.

### 4.5. Gas-Chromatography Analysis of Water Diesel-Derived Hydrocarbon Concentration and Biodegradation

GC-FID was used to evaluate the diesel biodegradation activity performed by *C. prolifera* during laboratory exposition and to evaluate the WAF in the three marine sites where *C. prolifera* thalli were collected. According to [96], 50 mL of contaminated seawater (with a diesel concentration of 1000 ppm) was added with the internal standard ortho-terphenyl (with a concentration of 50 ppm) and extracted (liquid–liquid extraction) with hexane (1:10 dilution of the solution of water and the standard). The organic fraction was evaporated to dryness, dissolved in 4 mL of hexane, and 1 μL of the obtained solution was analysed by gas-chromatography FID. GC–FID was performed using an Agilent Technologies 6890 N coupled to a FID detector, equipped with an HP5 column (30 m in length, 0.32 mm internal diameter, and 0.25 μm in thickness; Agilent Technologies, J&W Scientific Products, Santa Clara, CA). The carrier gas was helium, used at a flow rate of 1 mL min^−1^. The temperature was first set at 70 °C for 2 min and was increased to 230 °C at 20 °C min^−1^, then to 300 °C at 40 °C min^−1^ and finally set at 300 °C for 10 min. For the estimation of the biodegradation activity, the ratio between the n-C17 and the pristane peaks, which is considered a proxy of biodegradation, was used, comparing data collected at the beginning of the experiment (T_s_) and after two weeks (T_m_). Indeed, in samples collected at the end of experiment, the degradation of the C19 had already started, thus altering the considered ratio. To evaluate the concentration of diesel (ppm) in the water samples, total hydrocarbon concentrations were calculated, relating standard curves to peak areas highlighted by the chromatogram. The obtained values for the three marine sites were 31.99 mg L^−1^, 84.38 mg L^−1^ and 730.57 mg L^−1^ for Punta Don Diego, Isola Piana and Cala Finanza, respectively, thus confirming that the selected sites were suitable for sample collection in order to obtain data comparable with that of the laboratory experiment.

### 4.6. Statistical Analysis

Data regarding the functional traits of *C. prolifera*, the abundance of the bacterial community and the biodegradation activity were analysed by means of univariate statistical analyses with the software GMAV 5 and Past 4.11. Dependent Sample *t*–tests, ANOVAs and SNK tests were performed [97,98]. The composition of the bacterial community (OTUs) was analysed by means of multivariate statistical analysis with the software Past4.03, PermANOVA1.6 and PrimerV6 (PCA, PERMANOVA, SIMPER) [99,100,101,102]. A more detailed description of the statistics is available in the Supplementary Materials section. 

## 5. Conclusions

Overall, the results of this study proved that, at least within a defined range of diesel oil concentrations, *C. prolifera* tolerates and degrades seawater hydrocarbons, especially changing the community structure of its bacterial coating by enhancing the abundance of the selected *Vibrio* species. Therefore, this seaweed–bacteria association represents a promising native natural symbiosis for seawater phytoremediation of diesel hydrocarbons in Mediterranean costal environments subjected to small but prolonged diesel oil spills.

## Data Availability

All the research data are shareable by the authors if requested.

## Supplementary Materials

The following supporting information can be downloaded at https://www.mdpi.com/article/10.3390/plants12132507/s1, Table S1. Results of PERMANOVA on the controlled condition samples at T_s_, at T_f_ on the left, and on the field-collected samples on the right. Statistically significant results (*p* < 0.05) are highlighted in bold; Table S2. Results of the ANOVA tests to evaluate *C. prolifera* performance for the considered treatments (initial and final values for each functional trait); statistically significant results (*p* < 0.05) are given in bold. When *p* < 0.05, SNK post hoc tests were performed; Table S3. Results of the ANOVA tests to evaluate *C. prolifera* performance for the considered treatments (decrease during the experiment); statistically significant results (*p* < 0.05) are given in bold. When *p* < 0.05, SNK post hoc tests were performed; Table S4. Results of the ANOVA to test for differences in epiphytic bacterial abundances at the beginning (T_s_) and at the end (T_f_) of the experiment for each treatment ((0.01% *v*/*v* diesel; 1% *v*/*v* diesel; Control with no diesel). Statistically significant results (*p* < 0.05) are highlighted in bold. When *p* < 0.05, SKN post hoc test was also performed; Table S5. Results of ANOVAs and SNK post hoc tests to assess the differences in Vibrio2, Vibrio1, and Unclassified_Vibrionaceae8 abundances in the three T_f_ treatments. Statistically significant results (*p* < 0.05) are highlighted in bold; Table S6. Results of the two-way ANOVA to investigate the effect of the presence of *C. prolifera* (“Algae” factor, with two levels: “Yes” and “No”) in the C17/C19 ratio (used as proxy for biodegradation process) coupled with the experimental treatments (“Treatment” factor, with two levels: “0.01% *v*/*v*” and “1% *v*/*v*”). Statistically significant values (*p* < 0.05) are highlighted in bold. SNK post hoc test results are also showed for statistically significant results; Table S7. Results of the ANOVA to investigate the differences in bacterial α-diversity (expressed as number of OTUs) among experimental treatments. Statistically significant values (*p* < 0.05) are highlighted in bold. SNK post hoc test results are also showed for statistically significant results; Table S8. Results of the ANOVA to investigate the differences in bacterial β-diversity (expressed as Whittaker Index) among experimental treatments. Statistically significant values (*p* < 0.05) are highlighted in bold. SNK post hoc test results are also shown for statistically significant results; Table S9. Results of the ANOVA to investigate the differences in bacterial abundance of field-collected samples in the three sites, corresponding to three different diesel contamination levels. Statistically significant values (*p* < 0.05) are highlighted in bold. SNK post hoc test results are also shown for statistically significant results; Table S10. Results of the ANOVA to investigate the differences in bacterial α- and β-diversity (expressed as number of OTUs and as the Whittaker Index, respectively), of the field-collected samples among sites, corresponding to three different concentrations of diesel contamination.
