# Supplementary material for: Does Caulerpa prolifera with Its Bacterial Coating Represent a Promising Association for Seawater Phytoremediation of Diesel Hydrocarbons?
Source: Plants (Basel). 2023 Jun 30;12(13):2507. doi: 10.3390/plants12132507 (PMC10346704; doi:10.3390/plants12132507)
Supplement: Supplementary file 1 [file plants-12-02507-s001.zip › plants-2449343-supplementary.pdf]

Supplementary materials

Detail of the performed univariate and multivariate statistical analysis and extended results (for analysis with more than one factor)

Univariate statistical analysis

Cochran's tests were run prior to each ANOVA to test for homogeneity of variances and normality was assured by Kolmogorov-Smirnov test. Student–Newman–Keuls (SNK) tests were used for a posteriori comparison in case of significant ANOVA results, testing all the possible pairs of means computed on the considered groups [97].

The detail of the performed tests for each response variable are provided hereafter.

C. prolifera traits: ten one-way ANOVAs were on the whole run one for each response variable, to test for differences for each of them among the three different diesel concentration treatments (Treatment, 3 levels, fixed). In particular, for the number of blades, the total blade length and the total blade area, a separate analysis was performed for each sampling time, as data regarding the beginning (T_s) and the end of the experiment (T_f) were interdependent one from the other [97]. Moreover, three further ANOVAs were performed on data regarding the observed decrease for each trait recorded during the experiment (comparing initial and final data) for each treatment (Treatment, 3 levels, fixed). For mortality, instead, one only ANOVA was run, as mortality data were obviously refereed only at T_f (Treatment, 3 levels, fixed).

Table S1. Results of the ANOVA tests to evaluate C. prolifera *performance for the considered treatments (initial and final values for each functional trait); statistically significant results (P<0.05) are given in bold. When P<0.05, SNK post-hoc tests were performed.*

Source	*DF*	*Number of blades*				*Blade length*			
		T_s		T_f		T_s		T_f	
		F	P	F	p	F	P	F	P
Treatment	2	0.37	0.7046	1.25	0.3521	0.69	0.5374	2.98	0.1260
RES	6								
TOT	8								
Cochran's Test		C = 0.7524 *ns*		C = 0.654 *ns*		C = 0.5374 *ns*		C = 0.1260 *ns*	

Source	*DF*	*Blade area*				*Thalli mortality*		
		T_s		T_f				
		F	P	F	P	F	P	*SNK (Treatment)*
Treatment	2	1.30	0.3389	1.34	0.3297	8.25	**0.0189**	Control = 0.01% < 1%
RES	6							
TOT	8							
Cochran's Test		C = 0.615 *ns*		C = 0.7863 *ns*		C = 0.4902 *ns*		SE = 9.1639

Table S2. Results of the ANOVA tests to evaluate C. prolifera *performance for the considered treatments (decrease during the experiment); statistically significant results (P<0.05) are given in bold. When P<0.05, SNK post-hoc tests were performed.*

Source	*DF*	*Blade length*		*Blade area*		*Number of blades*	
		F	P		P		P
Treatment	2	6.54	0.0014	0.87	0.0024	0.56	0.0021
RES	6						

TOT	8			
Cochran's Test		C = 0.3421 *ns*	C = 0.7891 *ns*	C = 0.8926 *ns*
SNK (Treatment)				
		C<0.01%<1%	0.01%<C=1%	0.01%<C=1%
		SE = 0.0354	SE = 0.0678	SE = 0.0621

Bacterial abundance: one-way ANOVA was run to test for differences in the abundance of bacteria among the three different diesel concentration treatments (Treatments, 3 levels, fixed). Two ANOVAs were overall, performed one for each sampling time as data regarding the beginning (T_s) and the end of the experiment (T_f) were interdependent one from the other [97].

Table S3. Results of the ANOVA to test for differences in epiphytic bacterial abundances at the beginning (Ts) and at the end (Tf) of the experiment for each treatment ((0.01% v/v diesel; 1% v/v diesel; Control with no diesel). Statistically significant results (P<0.05) are highlighted in bold. When p<0.05, SKN post hoc test was also performed.

Source	*DF*	*Bacterial abundance*				
		T_s		*T_f*		*SNK (Treatment)*
		F	P	F	P	
Treatment	2	3.05	0.1220	47.31	**0.0002**	Control = 1% < 0.01%
RES	6					
TOT	8					
Cochran's Test		C = 0.6936 *ns*		C = 0.5449 *ns*		SE = 141249.89

Diversity indexes: Six one-way ANOVAs (Treatment, 3 levels, fixed) were performed (three for Alfa and three for Beta Diversity) considering both field and laboratory data (T_s and T_f).

Table S4. Results of the ANOVA to investigate the differences in bacterial α-diversity (expressed as number of OTUs) among experimental treatments. Statistically significant values (p < 0.05) are highlighted in bold. SNK post hoc test results are also showed for statistically significant results.

Source	*DF*	*α-diversity*				
		T_s		*T_f*		*SNK (Treatment)*
		F	P	F	P	
Treatment	2	8.81	0.055	9.64	**0.0494**	Control = 1% > 0.01%
RES	3					
TOT	5					
Cochran's Test		C = *ns*		C = *ns*		SE = 35.1714

Table S5. Results of the ANOVA to investigate the differences in bacterial β-diversity (expressed as Whittaker Index) among experimental treatments. Statistically significant values (p < 0.05) are highlighted in bold. SNK post hoc test results are also showed for statistically significant results.

Source	*DF*	*β-diversity*		
		T_s	*T_f*	*SNK (Treatment)*

		F	P	F	P	
Treatment	2	2.61	0.2206	41.12	**0,0066**	Control = 1% < 0.01%
RES	3					
TOT	5					
Cochran's Test		C = *ns*		C = *ns*		SE = 4.236

Relevant bacterial taxa abundance: Three one-way ANOVAs, one for the abundance of each taxon contributing more than 3.5% to the observed differences (in terms of dissimilarity) among diesel concentrations, were run (Treatment, 3 levels, fixed).

Table S6. Results of ANOVAs and SNK post hoc tests to assess the differences in Vibrio2, Vibrio1, and Unclassified_Vibrionaceae8 abundances in the three T_f treatments. Statistically significant results (p<0.05) are highlighted in bold.

Source	*DF*	*Vibrio2*		*Vibrio1*		*Unclassified_Vibrionaceae*	
		F	P	F	P	F	P
Treatment	2	14.01	**0.0301**	28.13	**0.0114**	8.46	0.0585
RES	3						
TOT	5						
		C = 0.9378		C = 7560		C = 0.8824	
		SNK (Treatment)					
		Control = 1% < 0.01%		0.01% < 1% = Control			
		SE = 0.0534		SE = 0.0354			

GC: Two two-way ANOVAs (due to interdependence of data, according to [97]) were run with data refereed to the laboratory experiment to test for differences in the C17-C19 ratio among diesel concentrations (Treatment, 2 levels, fixed and orthogonal) in tanks with and without *C. prolifera* (Algae, 2 levels, fixed and orthogonal) at T_s and T_m.

Table S7. Results of the two-way ANOVA to investigate the effect of the presence of C. prolifera *("Algae" factor, with two levels: "Yes" and "No") in the C17/C19 ratio (used as proxy for biodegradation process) coupled with the experimental treatments ("Treatment" factor, with two levels: "0.01% v/v" and "1% v/v"). Statistically significant values (p < 0.05) are highlighted in bold. SNK post hoc test results are also showed for statistically significant results.*

Source	*DF*	*C17/C19*

		T_s		T_m	
		F	P	F	P
Treatment	1	1.89	0.2069	7009.00	**0.0000**
Algae	1	1.80	0.2164	286.30	**0.0000**
Treatment x Algae	1	4.68	0.0625	166.82	**0.0000**
RES	8				
TOT	11				
Cochran's Test		C = 0.7215 *ns*		C = 0.3786 *ns*	
SNK (Treatment x Algae)					
Treatment					
0.01%		**Yes < No ****			
1%		**Yes < No ***			
Algae					
Yes		**0.01% < 1% ****			
No		**0.01% < 1% ****			
SE = 0.0058					

Table S8. Results of the ANOVA to investigate the differences in bacterial abundance of field-collected samples in the three sites, corresponding to three different diesel contamination levels. Statistically significant values (p < 0.05) are highlighted in bold. SNK post hoc test results are also showed for statistically significant results.

Source	*DF*	*Bacterial abundance*		
		F	P	*SNK (Treatment)*
Treatment	2	22.19	**0.0017**	Low > High = Control
RES	4			
TOT	6			
Cochran's Test		C = *ns*		SE = 48073.33

Table S9. Results of the ANOVA to investigate the differences in bacterial α- and β-diversity (expressed as number of OTUs and as the Whittaker Index, respectively) of the field-collected samples among sites, corresponding to three different concentrations of diesel contamination.

Source	DF	Bacterial diversity			
		α-diversity		β-diversity	
		F	P	F	P
Treatment	2	2.98	0.194	12.86	0.0638
RES	3				
TOT	5				
Cochran's Test		C = *ns*		C = *ns*	

Multivariate statistical analysis

Multivariate analyses were based on Hellinger distance, which depends on the differences in OTU proportion between samples, decreases the importance of OTU abundance over their occurrence and avoids the double-zero problem when comparing OTU composition between samples [99,100]. A Principal Component Analysis (PCA) was performed on all samples to visualize data distribution.

A distance-based permutational multivariate analysis of variance (PERMANOVA) [101] was then performed to test for differences in the genus composition of the bacterial community in relation to diesel concentration both in the laboratory and in the field experiment (Treatment, 3 levels, fixed) (PERMANOVA). The analyses were based on Bray-Curtis dissimilarities calculated on normalized data (Hellinger). Each term in the analysis was tested using 9999 random permutations. Regarding laboratory data, due to data interdependence [98], two PERMANOVAs were performed on data (at the genus level) collected at T_s and T_f respectively. For field data, instead, one PERMANOVA was performed, as only one dataset was present. Where significant results were highlighted by the PERMANOVA, significant terms relevant to the hypotheses were investigated through post hoc pair-wise comparisons and a SIMPER test was also run (Primer v6) to point out the relative contribution of each taxon to the dissimilarities observed among treatments [102]. Moreover, to investigate the differences among diesel concentrations evidenced by the SIMPER test for the main bacterial taxa, a one-way ANOVA was also run for each relevant taxon abundance, as described hereafter.

Furthermore, a principal component analysis (PCA) was performed summarizing the variation, in terms of genus composition, among 1) diesel concentrations at the end of the experiment (for which the PERMANOVA provided significant results) for laboratory data and 2) sites for field data (Past). Finally, the Species Richness Index and the Whittaker Index were used to evaluate Alfa and Beta Diversity respectively on laboratory and field data regarding the genus composition of bacterial communities (considering field data and both T_s and T_f for laboratory data) and different ANOVAs were run to test for differences in such indexes among treatments, ad described hereafter.

Table S10. Results of PERMANOVA on the controlled conditions samples at T_s, at T_f on the left, and on the field-collected samples on the righ. Statistically significant results ($p<0.05$) are highlighted in bold.

Source	*DF*	T_s		T_f		*Field samples*	
		F	*p(perm)*	*F*	*p(perm)*	*F*	*P(perm)*
Treatment/Site	2	22.614	0.0702	57.278	**0.021**	22.602	0.0665
RES	3						
TOT	5						
